# Current News

**Published:** 1889-09

**Authors:** 


### Current News.

The International Tooth Crown Company have brought a civil suit for sixty thousand dollars and six months' imprisonment against Dr. J. N. Crouse for interfering with their business.

---

It having been decided that it would not be the right thing to do to use the funds of the American Dental Protective Association for the defence of Dr. Crouse, the matter was brought to the notice of the members of the American Dental Association, when one thousand dollars was promptly voted from the treasury for Dr. Crouse's defence; a private purse, amounting to fully two thousand dollars more, was also quickly subscribed by members present. The subscription is still open, and names may be sent to this office.

---

The International Tooth Crown Company could not have done anything that would have tended more to rouse the profession than the suit instigated against Dr. Crouse, which virtually amounts to persecution of an honest man, who has taken upon himself the responsibility of the defence of the profession. You cannot do less than send in your membership fee to support him, especially when it will only be used to defend *yourself* from the oppression of the newly revised "Rubber Company."

---

The following resolutions were unanimously passed by the American Dental Association:

"In view of the injustice which the profession has sustained in the past, as well as the annoyances to which it may be subjected in the future, and fully appreciating the arduous and important labors of Dr. J. N. Crouse, of Chicago, be it

"*Resolved*, That Dr. Crouse is entitled to the earnest and practical support of every dentist in the United States; and further, be it

"*Resolved*, That the American Dental Association fully approves of the formation, the plans, and methods of the Dental Protective Association of the United States, and pledge to it our united and continued support and moral aid."

We are informed that the office and practice of the late Dr Vanderplant is for sale at a decided bargain. See advertising pages for notice.

---

Hayden Dental Society of Chicago.—The Hayden Dental Society was organized and incorporated under the laws of the State of Illinois, August 3, 1889.

The object of the society is the professional and social improvement of its members. Meetings will be held in Chicago on the third Monday of each month (except July and August).

The following officers were elected for the ensuing year: President, Louis Ottofy; Vice-President, A. W. Freeman; Secretary, A. J. Oakey. Board of Directors: J. W. Rogers, J. L. Ubellar, H. P. Smith. A. J. Oakey, *Secretary.*

---

### Abstract of a Bill for an Act to Regulate the Practice of Dentistry in the State of Minnesota.

*Be it enacted by the Legislature of the State of Minnesota.*

Section 1. From and after September 1, 1889, it shall be unlawful for any person to practise dentistry in this State, unless he shall first have obtained a certificate of registration thereto, and filed the same or a certified copy thereof with the clerk of the district court of the county of his residence, all as hereinafter provided.

Sec. 2. A board of examiners, to consist of five resident practising dentists, is hereby created, whose duty it shall be to carry out the purposes and enforce the provisions of this act. The members of the first board under the provisions of this act shall consist of the members of the present board of dental examiners, existing under chapter 199 of the General Laws of 1885, who shall hold their offices as members of such new board for the term for which they were appointed under said former act, and until their successors are duly appointed. All vacancies in said board shall be filled by appointment by the governor as hereinafter provided. The term for which members of said board shall be appointed shall be three years, and until their successors shall be duly appointed. It is also hereby provided that no person shall serve to exceed two terms in succession. In case of any vacancy occurring in said board in the term of any member of said board, such vacancy shall be filled for such unexpired term by the governor from names to be presented to him within two months of the occurrence of such vacancy by the Minnesota State Dental Association in the same manner as hereinafter provided. It shall be the duty of said Minnesota State Dental Association after September 1, 1889, annually prior to August 10 to present to the governor the names of twice as many practising dentists resident in this State as there are regular members to be appointed of said board prior to September in the following year. All appointments by the governor shall be made within twenty days of the submission of such names to him, and if such names shall hot be submitted to him within the allotted time, he shall make his appointments within twenty days from the expiration of the time allotted for such presentation of names from among the resident practising dentists. *Provided*, That nothing in this act shall prevent the appointment of two members of said board from among the resident practising dentists not members of said Minnesota State Dental Association, if the governor shall so elect.

Sec. 5. Any person or persons who shall desire to begin the practice of dentistry in the State of Minnesota on and after September 1, 1889, shall file his name, together with an application for examination, with the secretary of the State Board of Dental Examiners, and at the time of making such application shall pay to the secretary of said board a fee of ten dollars, and shall present himself at the first regular meeting thereafter of said board to undergo examination before that body. In order to be eligible for such examination such person shall present to said board his diploma from some dental college in good standing, and shall give satisfactory evidence of his rightful possession of the same, provided also that the board may in its discretion admit to examination such other persons as shall give satisfactory evidence of having been engaged in the practice of dentistry ten years prior to the date of passage of this act. Said board shall have the power to determine the good standing of any college or colleges from which such diplomas may have been granted. The examinations shall be elementary and practical in character, but sufficiently thorough to test the fitness of the candidate to practise dentistry. It shall include, written in the English language, questions on the following subjects : Anatomy, physiology, chemistry, materia medica, therapeutics, metallurgy, histology, pathology, operative and surgical dentistry, mechanical dentistry, and also demonstrations of their skill in operative and mechanical dentistry. All persons successfully passing such examinations shall be registered as licensed dentists in the board register provided for in Section 4, and also receive a certificate of such enregistration, said certificate to be signed by the president and secretary of the board. The examination fee shall in no case be refunded.

Sec. 6. Recipients of said certificate of enregistration shall present the same for record to the clerk of the district court of the county in which they reside, and shall pay a fee of fifty cents to said clerk for the registration of the same. Said clerk shall record said certificate in a book to be provided by him for that purpose. Any person so licensed removing his residence from one county to another in this State, before engaging in the practice of dentistry in such other county shall obtain from the clerk of the district court of the county in which said certificate of registration is recorded a certified copy of such record, or else obtain a new certificate of registration from the board of examiners, and shall, before commencing practice in such county, file the same for record with the clerk of the court of the county to which he removes, and pay the clerk for recording the same the fee of fifty cents. Any failure, neglect, or refusal on the part of any person holding such certificate or copy of record to file the same for record as hereinbefore provided for six months from the issuance thereof shall forfeit the same. Such board shall be entitled to a fee of one dollar for the reissue of any certificate, and the clerk of the district court for any county shall be entitled to a fee of one dollar for making and certifying a copy of the record of any such certificate.

SEC. 7. All persons shall be said to be practising dentistry within the meaning of this act who shall for a fee or salary, or other reward paid either to himself or to another person for operations or parts of operations of any kind, treat diseases or lesions of the human teeth or jaws or correct malpositions thereof. But nothing in this act contained shall be taken to apply to acts of *bona fide* students of dentistry, done in the pursuit of clinical advantages under the direct supervision of a preceptor or a licensed dentist in this State during the period of their enrolment in a dental college, and attendance upon a regular uninterrupted course in such college.

SEC. 9. Any person who shall violate any of the provisions of this act shall be deemed guilty of a misdemeanor, and upon conviction may be fined not less than twenty dollars nor more than one hundred dollars, or to be confined not less than one month nor more than three months in the county jail, or both. And all fines thus received shall be paid into the common school fund of the county in which such conviction takes place.

SEC. 10. Any person who shall knowingly or falsely claim or pretend to have or hold a certificate of enregistration, diploma, or degree granted by a society or by said board, or who shall falsely and with the intent to deceive the public, claim or pretend to be a graduate from any incorporated dental college, not being such graduate, shall be deemed guilty of a misdemeanor, and shall be liable to the penalties provided in Section 9 of this act.

SEC. 11. Justices of the peace and the respective municipal courts shall have jurisdiction over violations of this act. It shall be the duty of the respective county attorneys to prosecute all violations of this act.

SEC. 13. Every registered dentist shall in each and every year after 1889 pay to said Board of Examiners the sum of one dollar as a license fee for such year. Such payment shall be made prior to May 1 in each and every year, and in case of default in such payment by any person, his certificate may be revoked by the Board of Examiners upon twenty days' notice of the time and place of considering such revocation. But no license shall be revoked for such non-payment if the person so notified shall pay before or at such consideration his fee and such penalty as may be imposed by said Board. *Provided*, That said Board may impose a penalty of five dollars and no more on any one so notified as a condition of allowing his license to stand. *Provided further*, That said Board of Examiners may collect any such dues by suit.

